# Exploring prior diseases associated with incident late-onset Alzheimer’s disease dementia

**DOI:** 10.1371/journal.pone.0228172

**Published:** 2020-01-24

**Authors:** Jung-Yu Liao, Charles Tzu-Chi Lee, Tsung-Yi Lin, Chin-Mei Liu

**Affiliations:** 1 Department of Health Promotion and Health Education, National Taiwan Normal University, Taipei, Taiwan; 2 Department of Marketing and Distribution Management, Hsing Wu University, New Taipei City, Taiwan; 3 Taiwan Centers for Disease Control, Taipei, Taiwan; Nathan S Kline Institute, UNITED STATES

## Abstract

Studies have identified prior conditions associated with late-onset Alzheimer’s disease dementia (LOAD), but all prior diseases have rarely been screened simultaneously in the literature. Our objective in the present study was to identify prior conditions associated with LOAD and construct pathways for them. We conducted a population-based matched case-control study based on data collected in the National Health Insurance Research database of Taiwan and the Catastrophic Illness Certificate database for the years 1997–2013. Prior diseases definitions were based on the first three digits of the codes listed in the International Classification of Diseases, Ninth Revision, Clinical Modification (ICD-9-CM). Inclusion criteria required that each ICD-code existed for at least 1 year and incurred at least 2 outpatient visits or inpatient diagnosis. The case group comprised 4,600 patients newly diagnosed with LOAD in 2007–2013. The LOAD patients were matched by sex and age to obtain 4,600 controls. Using stepwise multivariate logistic regression analysis, diseases were screened for 1, 2 …, 9 years prior to the first diagnosis of LOAD. Path analysis was used to construct pathways between prior diseases and LOAD. Our results revealed that the following conditions were positively associated with the incidence of LOAD: anxiety (ICD-code 300), functional digestive disorder (ICD code 564), psychopathology-specific symptoms (ICD-code 307), disorders of the vestibular system (ICD-code 386), concussion (ICD-code 850), disorders of the urethra and urinary tract (ICD-code 599), disorders of refraction and accommodation (ICD-code 367), and hearing loss (ICD-code 389). A number of the prior diseases have previously been described in the literature in a manner identical to that in the present study. Our study supports the assertion that mental, hearing, vestibular system, and functional digestive disorders may play an important role in the pathogenesis of LOAD.

## Introduction

Dementia is a severely debilitating health condition that imposes burdensome social and economic costs, and rapidly aging populations have increased the prevalence of dementia worldwide [1]. Treatments for dementia can help only to relieve symptoms; i.e., there is no cure. Recognizing the risk of dementia is a critical issue for preventing dementia prevalent.

Late-onset Alzheimer’s disease dementia (LOAD) accounts for 60–80% of all cases of dementia [2]; however, the etiology of LOAD has not yet been verified. LOAD is currently regarded as a neurodegenerative disease, wherein nearly 70% of the risk factors have a genetic link [3, 4]. Nonetheless, epidemiological studies have demonstrated that many diseases are associated with the incidence of LOAD. Vascular risk factors [5–8], including diabetes, hypertension, heart disease, obesity, and neuropsychiatric symptoms (e.g., depression, anxiety, and stress), have been examined in the context of cognitive decline [9–13]. Structural brain abnormalities are commonly observed in people with affective disorders, including hippocampal atrophy in patients with a history of depression [10, 14]. Major depression may be a risk factor for the development of AD, and patients with lifelong depression have a two-fold higher likelihood of developing AD and exhibiting more AD-related neuropathological symptoms [12, 13]. Pathological manifestation in head injury have also been linked to the risk of LOAD, including increased amyloid-beta and tau pathology, neuroinflammation, and axonal and cytoskeletal changes in the brain [15–17]. Hearing loss has been reported as a risk factor for dementia [18–21], where even mild levels suggest a link [22, 23]. Patients with hearing loss may suffer from social isolation, depression, disability, and increased risk of dementia [24–27]. Moreover, recent studies in animals have demonstrated that the microbiome-gut-brain axis may be involved in the pathogenesis of Alzheimer’s disease [28–30].

Identifying potentially prior diseases is crucial to the prevention of LOAD epidemics. To our knowledge, there has been a dearth of research on associations among all diseases and LOAD simultaneously. LOAD is influenced by multiple factors, and it is essential to explore the relevant factors using different research methods. The present study aims to determine prior diseases associated with LOAD simultaneously, and construct pathways analysis for these diseases associated with LOAD.

## Materials and methods

### Ethics statement

Approval for this study was obtained from the Institutional Review Board (IRB) of National Taiwan Normal University (Protocol Number: 201712HM015). Written consent was exempted because the data was obtained from the National Health Insurance Research Database (NHIRD) of Taiwan, which contains de-identified secondary data released for research purposes.

### Data sources

We used data files pertaining to outpatient care, inpatient care, ambulatory care, and details of prior medical conditions from the NHIRD in Taiwan for the years 1997–2013 [31]. We also used the Catastrophic Illness Certificate (CIC) database of Taiwan for the same period. The insurance system records all patients with 30 categories of catastrophic illness, including malignant neoplasm, uremia, and chronic psychotic disorders (e.g., dementia) with the Registry of Patients with Catastrophic Illness. The attending physician of any patient diagnosed with a catastrophic illness can submit relevant information to the CIC. A committee formally reviews the applications, and if approved, patients are then exempted from co-payment during the effective period in Taiwan. Thus, this data can be regarded as comprehensive.

### Study design and population

**Fig 1:**
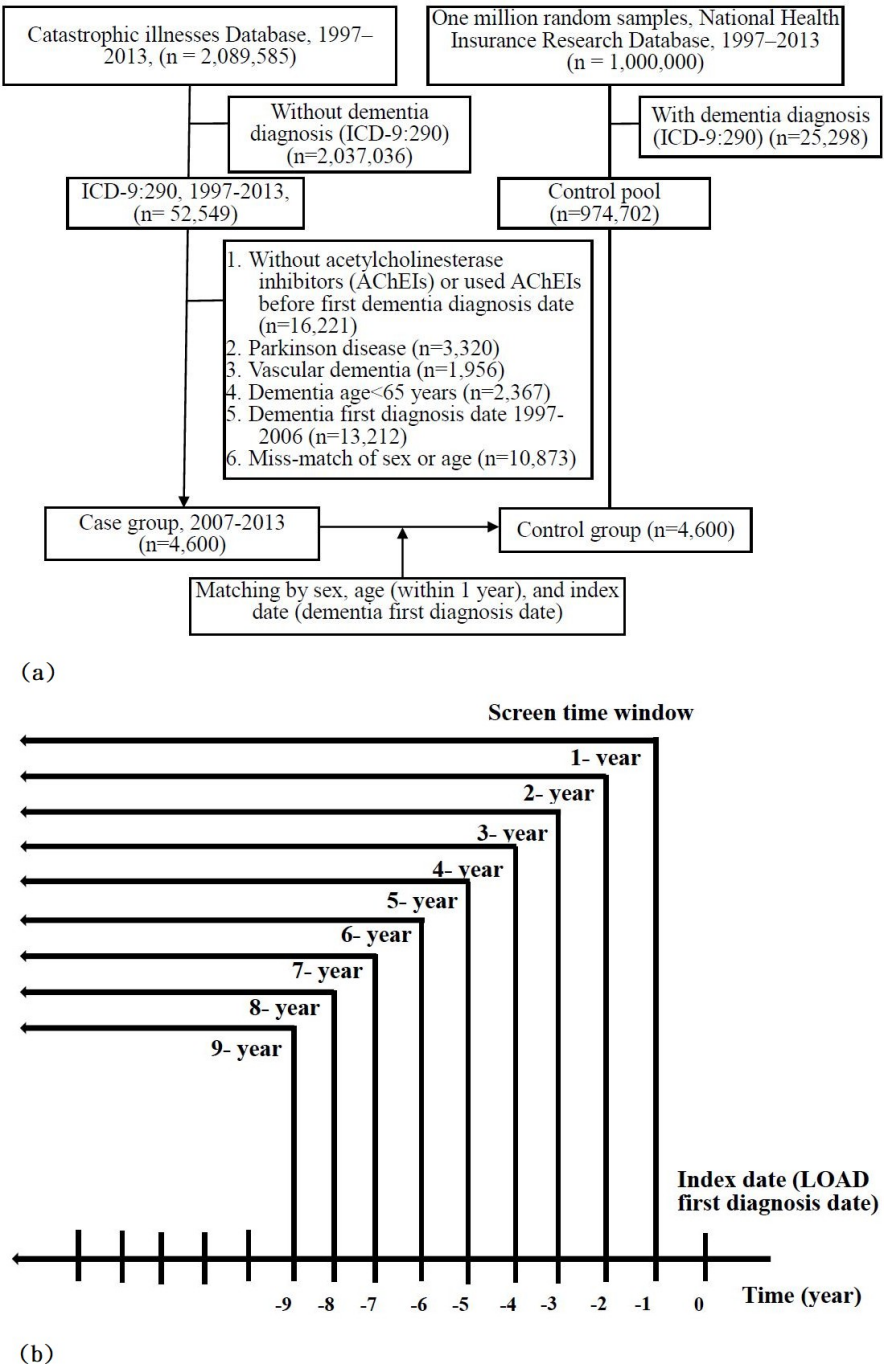
(a) Flowchart of subject selection process; (b) screen time windows used to identify prior diseases associated with LOAD.

In this population-based matched case-control study, LOAD cases were defined as CIC patients newly diagnosed with dementia (International Classification of Diseases, Ninth Revision, Clinical Modification, ICD-9-CM: 290.xx) in 2007–2013 and prescribed any acetylcholinesterase inhibitors (AChEIs) (Anatomical Therapeutic Chemical, [ATC] N06D) after the LOAD diagnosis date. We excluded the following patients: (1) those who did not use AChEIs or had used AChEIs before the LOAD diagnosis date, (2) those diagnosed with Parkinson's disease (PD, ICD-9 332.0, 332.1), (3) those diagnosed with Vascular dementia (VaD, ICD-9 290.40, 290.41, 290.42, 290.43), (4) those aged <65 years, and (5) those with a first diagnosis date of LOAD in the 1997–2006 period. The index date was defined as the first date on which the patient received a definitive diagnosis of LOAD (Fig 1). The case group contained 4,600 patients newly diagnosed with LOAD in 2007–2013. LOAD patients were matched by sex and age to obtain 4,600 controls.

Prior diseases definitions were based on the first three digits of the codes listed in the International Classification of Diseases, Ninth Revision, Clinical Modification (ICD-9-CM). Inclusion criteria required that each ICD-code existed for at least 1 year and incurred at least 2 outpatient visits or inpatient diagnosis. Prior diseases associated with LOAD were screened out for 1, 2, 3, …,9 years before the date of first diagnosis LOAD (Fig 1.). All medical claims between 1997 and 2013 containing this code were obtained from the NHIRD for further analysis. Finally, we constructed pathways related to the identified prior diseases and plotted the relationships among diseases.

### Statistical analysis

The Chi-square test was used to compare the distributions of demographic factors of patients newly diagnosed with LOAD and controls. Associated diseases were identified using a conditional logistic regression model. Stepwise multivariate logistic regression was used to identify the diseases associated with LOAD and the adjusted odds ratio (OR). The 95% confidence was to set a P value of < 0.05. Two-sided data analysis was performed using the statistical package SAS 9.4 (SAS Institute Inc., Cary, NC, USA).

Path analysis correction was used with significant diseases during 4-year periods to construct pathways from diseases to LOAD. Five statistical tests were used to evaluate the overall goodness-of-fit of the correction model, including standardized root mean square residual (SRMR), root mean square error of approximation (RMSEA), comparative fit index (CFI), goodness-of-fit statistic (GFI), and normed-fit index (NFI) [32].

We fitted a hypothesized pathway model to the LOAD and control groups, and then executed a correction model to establish positive and negative associations via modification indices (MI). Finally, the positive and negative correction models were combined into one model using MIs for correction. The correction procedure modified the initial model one path at a time with the aim of improving the goodness-of-fit. Candidate paths where MI > 0 were then added to the correction model. The final step involved systematically trimming non-significant pathways based on coefficient estimates with a P value of > 0.05. In each step, interim evaluations of MI were conducted to identify relevant pathways that arose after simplifying the model. We included in the final correction model only the significant paths (P < 0.05) for which there was an acceptable overall goodness-of-fit. The overall process was stopped when no additional significant pathways were suggested by the MI. The direct and indirect effects of prior diseases on LOAD incidence were defined using the model constraint procedure and the maximum likelihood robust estimator. Total effects (determined as the sum of direct effects and indirect effects) were also calculated. Data analysis was performed using AMOS 21 (IBM Corp., 2012). Similar analysis was used in our previous research on diseases correlating with amyotrophic lateral sclerosis [33].

## Results

### Sample characteristics

The two groups comprised 4,600 patients newly diagnosed with LOAD (≥ 65 years of age) and 4,600 sex- and age-matched control groups (Table 1). Table 1 presents a comparison of the characteristics of the two groups. Most of the patients with LOAD were female (63.3%), the diagnosis age range was predominantly 65–79 years (68.43%), and half of the patients’ insurance premiums belonged to the fixed premium and dependent groups (49.33%). Patients who were insured under agriculture and fisheries categories had a lower incidence of LOAD.

**Table 1:** Characteristics of patients with and without LOAD.

Characteristics	LOAD, (N = 4,600)	Without LOAD, (N = 4,600)	*P* value
Gender N (%)			
Female	2,912 (63.30)	2,912 (63.30)	Exact match
Male	1,688 (36.70)	1,688 (36.70)	
Age of diagnosis (year) N (%)			
65–79	3,148 (68.43)	3,154 (68.57)	Exact match
≥ 80+	1,452 (31.57)	1,446 (31.43)	
Insurance premiums, NT$			
Low income	12 (0.26)	10 (0.22)	<0.001
Fixed premiums or dependent	2,269 (49.33)	1,867 (40.59)	
< 20,000	967 (21.02)	1,070 (23.26)	
20,000~39,999	1,333 (28.98)	1,623 (35.28)	
≥ 40,000	19 (0.41)	30 (0.65)	
Job			
Government employees	27 (0.59)	18 (0.39)	<0.001
Employees of private enterprises	68 (1.48)	68 (1.48)	
Agriculture and fisheries	1,267 (27.54)	1,570 (34.13)	
Low-income with social welfare	993 (21.59)	1,082 (23.52)	
Unemployed	2,245 (48.80)	1,862 (40.48)	
Alzheimer drugs N (%)			
No	0 (0.00)	4,600 (100.00)	
Yes	4,600 (100.00)	0 (0.00)	

### Association between prior diseases and incidence of LOAD

We identified a total of 39 prior conditions that were significantly associated with the incidence of LOAD (1-year periods), which included 23 positive and 16 negative associations (Table 2). Conditions that occurred eight years prior to the first diagnosis included episodic mood disorders (ICD-code 296), concussion (ICD-code 850), fracture of radius and ulna (ICD-code 813), anxiety (ICD-code 300), local infections of skin and subcutaneous tissue (ICD-code 686), functional digestive disorders (ICD-code 564), vertiginous syndrome and disorders of vestibular system (ICD-code 386), and diabetes mellitus (ICD-code 250). Conditions with an early negative effect on LOAD incidence included asthma (ICD-code 493), disorders of synovium, tendon, and bursa (ICD-code 727), and ill-defined sprains and strains (ICD-code 848). General medical examination (ICD-code V70) and prophylactic vaccination (ICD-code V04) were also significant associated with LOAD and were therefore included in the path analysis; i.e., to determine whether they would have an impact on the model.

**Table 2:** Prior diseases associated with LOAD as a function of screen time windows prior to diagnosis.

ICD-code	Disease	1- to 9- years before LOAD first diagnosis OR^a^								
		1-	2-	3-	4-	5-	6-	7-	8-	9-
331	Other cerebral degenerations	6.8	4.8							
311	Depressive disorder	2.1	1.9	1.7	1.8					
296	Episodic mood disorders	2.1	1.9	1.9	1.7	1.9	1.9	1.9	2.1	
783	Symptoms concerning nutrition, metabolism, and development	1.6								
850	Concussion	1.5	1.4	1.6	1.6	1.5	1.7	1.7	1.7	
924	Contusion of lower limb	1.4	1.4	1.4	1.3	1.3	1.2	1.3		
873	Other open wound of head	1.4	1.4	1.4	1.4	1.4				
813	Fracture of radius and ulna	1.4	1.7	1.7	2	1.8	1.8	1.8	1.9	1.9
389	Hearing loss	1.4	1.4	1.3	1.4	1.4	1.4			
300	Anxiety, dissociative and somatoform disorders	1.4	1.4	1.3	1.4	1.3	1.3	1.3	1.2	1.3
686	Other local infections of skin and subcutaneous tissue	1.3	1.3	1.3	1.3	1.2	1.2	1.3	1.3	1.3
599	Other disorders of urethra and urinary tract	1.3	1.3	1.3	1.3	1.3	1.3	1.2		
578	Gastrointestinal hemorrhage	1.3			1.4	1.4				
564	Functional digestive disorders	1.3	1.4	1.3	1.3	1.2	1.2	1.2	1.1	
486	Pneumonia	1.3	1.2							
437	Other and ill-defined cerebrovascular disease	1.3	1.3	1.2	1.3	1.3	1.3	1.3		
307	Special symptoms or syndromes, not elsewhere classified	1.3	1.3	1.2	1.2	1.2				
V70	General medical examination	1.2	1.2	1.2	1.2	1.3	1.3	1.3	1.2	1.2
V04	Need for prophylactic vaccination and inoculation against certain diseases	1.2	1.2	1.2	1.1	1.2	1.2	1.1	1.2	1.2
733	Other disorders of bone and cartilage	1.2		1.1		1.2	1.2			
682	Other cellulitis and abscess	1.2								
386	Vertiginous syndromes and other disorders of vestibular system	1.2	1.2	1.2	1.2	1.2			1.2	1.2
367	Disorders of refraction and accommodation	1.2	1.2	1.2	1.2		1.3			
692	Contact dermatitis and other eczema	1.1	1.2	1.1	1.1					
250	Diabetes mellitus	1.1					1.2	1.2	1.2	1.3
729	Other disorders of soft tissues	0.9	0.9	0.9						
571	Chronic liver disease and cirrhosis	0.9	0.9			0.9	0.9			
848	Other and ill-defined sprains and strains	0.8	0.8	0.8	0.8	0.8	0.8	0.8	0.7	0.7
847	Sprains and strains of other and unspecified parts of back	0.8								
785	Symptoms involving cardiovascular system	0.8	0.8	0.8						
726	Peripheral enthesopathies and allied syndromes	0.8	0.9							
493	Asthma	0.8	0.8	0.8	0.8	0.8	0.8	0.8	0.8	0.8
477	Allergic rhinitis	0.8	0.9							
413	Angina pectoris	0.8	0.8	0.8	0.8	0.8				
41	Bacterial infection in conditions classified elsewhere and of unspecified site	0.8								
V45	Other postprocedural states	0.7	0.7							
727	Other disorders of synovium, tendon, and bursa	0.7	0.7	0.7	0.6	0.7	0.7	0.7	0.7	0.7
597	Urethritis, not sexually transmitted, and urethral syndrome	0.7	0.7							
463	Acute tonsillitis	0.7	0.8	0.8	0.8	0.8				
518	Other diseases of lung	0.6	0.6	0.6	0.5	0.6				
273	Disorders of plasma protein metabolism	0.5								
217	Benign neoplasm of breast	0.5	0.6	0.5	0.5					

^a^ Odds-ratios (OR) with *P* value < 0.05 are shown in this table.

### Effects of prior diseases on LOAD incidence

The goodness-of-fit statistics revealed that the final correction models were acceptable (S1 Table). Table 3 presents the prior diseases with total, direct, and indirect effects on LOAD incidence for the 4-year period prior to diagnosis, after controlling for general medical examinations (ICD-code V70). Total effects exceeding 0.037 were identified in eight of the prior diseases, including anxiety, dissociative, and somatoform disorders (ICD-code 300); functional digestive disorders (ICD-code 564), special symptoms or syndromes not classified elsewhere (ICD-code 307); disorders of the vestibular system (ICD-code 386); concussion (ICD-code 850); disorders of the urethra and urinary tract (ICD-code 599); disorders of refraction and accommodation (ICD-code 367); and hearing loss (ICD-code 389). Two diseases with total negative effects were other diseases of the lungs (ICD-code 518) and other disorders of the synovium, tendon, and bursa (ICD-code 727).

**Table 3:** Total, direct, and indirect effects of significant prior diseases on LOAD incidence in the 4-year period prior to date of diagnosis.

ICD-code	Disease	Total effect^a^	Direct effect^a^	Indirect effect^a^
300	Anxiety, dissociative and somatoform disorders	0.089	0.038	0.051
564	Functional digestive disorders	0.060	0.032	0.028
307	Special symptoms or syndromes, not elsewhere classified	0.053	0.047	0.006
386	Vertiginous syndromes and other disorders of vestibular system	0.052	0.031	0.021
850	Concussion	0.050	0.046	0.004
599	Other disorders of urethra and urinary tract	0.044	0.030	0.014
367	Disorders of refraction and accommodation	0.042	0.040	0.001
389	Hearing loss	0.037	0.037	0.000
692	Contact dermatitis and other eczema	0.035	0.024	0.011
686	Other local infections of skin and subcutaneous tissue	0.035	0.036	-0.001
437	Other and ill-defined cerebrovascular disease	0.033	0.023	0.010
813	Fracture of radius and ulna	0.028	0.028	0.000
311	Depressive disorder	0.027	0.026	0.001
413	Angina pectoris	0.009	0.000	0.009
463	Acute tonsillitis	0.009	0.000	0.009
493	Asthma	0.008	0.000	0.008
296	Episodic mood disorders	0.007	0.000	0.007
873	Other open wound of head	0.004	0.000	0.004
848	Other and ill-defined sprains and strains	0.002	0.000	0.002
924	Contusion of lower limb and of other and unspecified sites	0.000	0.000	0.000
727	Other disorders of synovium, tendon, and bursa	-0.029	-0.046	0.016
518	Other diseases of lung	-0.030	-0.030	0.000
V70	General medical examination	0.051	0.037	0.014

^a^ Total effect reflects an association between prior diseases and LOAD incidence via all paths in the model; an indirect effect reflects this association minus the direct effect of any path from a prior disease to LOAD incidence; and a direct effect is simply the total effect minus the total indirect effect.

### Pathway between relevant prior diseases and incidence of LOAD

**Fig 2:**
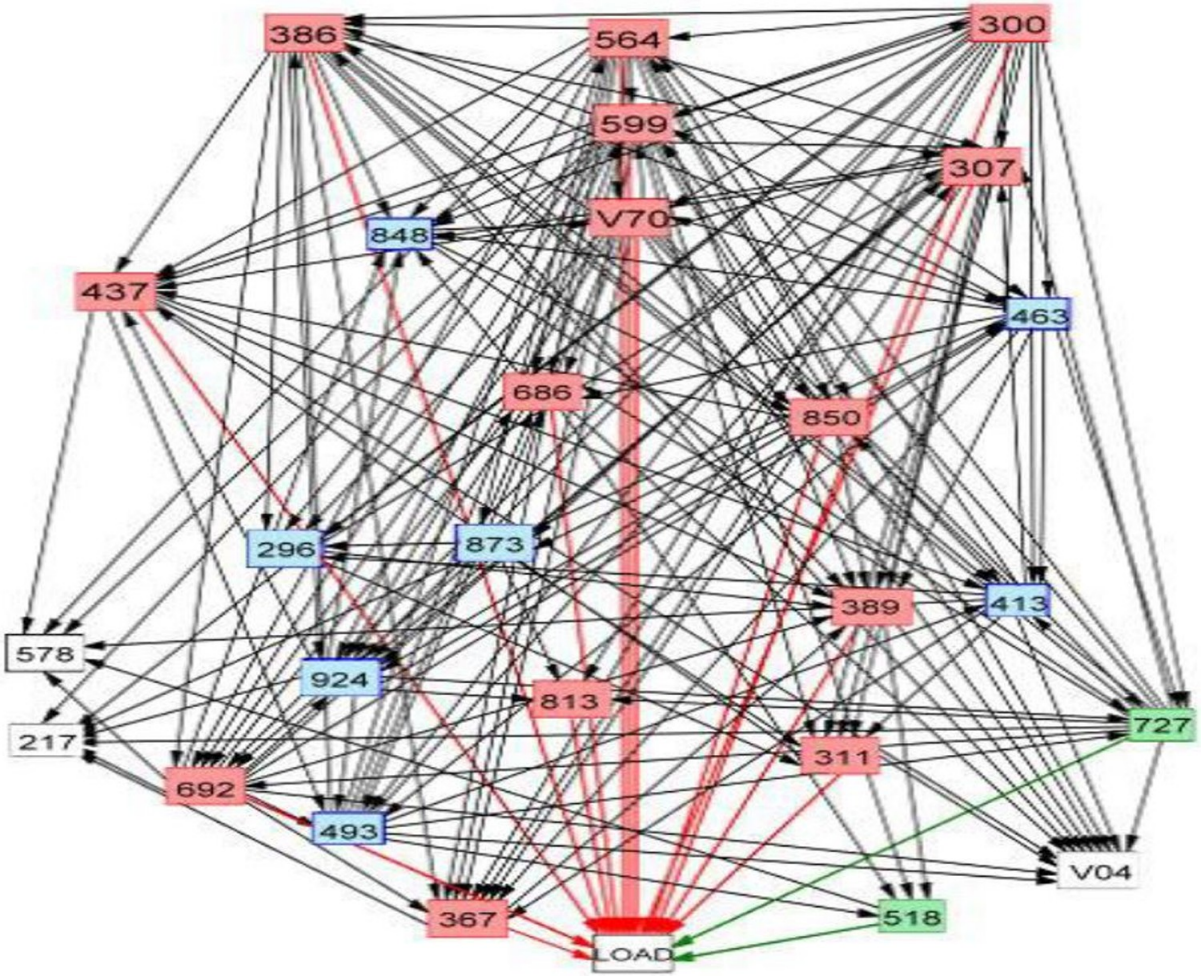
Final path analysis model for diseases associated with LOAD in the 4 years prior to the first diagnosis of LOAD. The red and blue lines respectively indicate direct positive and negative links between prior diseases and LOAD. Codes listed in the International Classification of Diseases, Ninth Revision, are displayed in the box.

Fig 2 presents the final pathways model of total effects (merging positive and negative effects) on LOAD incidence for the 4-year period prior to the first diagnosis of LOAD (S1 Fig: positive final pathways model; S2 Fig: negative pathway model). Two features of the final model are noteworthy. First, five diseases (ICD-codes 300, 564, 599, 386, and 850) in upper positions in the final model have numerous direct pathways to other diseases in the model. Second, mental disorders (ICD-codes 300, 307, 311) play a predominant role in the model in terms of total positive effects on LOAD incidence.

## Discussion

This is the first nationwide study in which multiple prior diseases linked to LOAD were simultaneously identified using stepwise multivariate logistic regression and path analysis. In the path analysis model, the following conditions had significant positive effects on LOAD incidence: anxiety (ICD-code 300), functional digestive disorders (ICD-code 564), special symptoms or syndromes not classified elsewhere (ICD-code 307), disorders of the vestibular system (ICD-code 386), concussion (ICD-code 850), disorders of the urethra and urinary tract (ICD-code 599), disorders of refraction and accommodation (ICD-code 367), and hearing loss (ICD-code 389).

Mental diseases have previously been identified as a risk factor for AD [7, 34], as a remote risk factor [35] and as a proximal prodromal feature of AD [36]. One Finnish study [36] similar to this work investigated the link between mental and behavioral disorders and AD. They found that depression and other mood disorders were associated with the risk of AD within a 5-year time window but not within a 10-year time window. The associations between mental/behavioral disorders and AD were modest and dependent on the time window near the onset. However, we found those mental diseases (ICD-codes 300, 311, 296) may reach associated with the risk of LOAD around 6- to 9- years period prior to the first diagnosis of LOAD. Our study differed from that study in two fundamental ways. The database used in this work links inpatient and outpatient medical records, was compiled more recently, and is more comprehensive. Furthermore, our model analyses each ICD-code as a variable rather than as a cluster of ICD-codes, thereby reducing the possibility of misdiagnosing mental disorders.

Our results revealed that vertiginous syndromes and disorders of the vestibular system (ICD-code 386) have a positive influence on LOAD incidence. It has been reported that bilateral vestibulopathy is due to a dysfunction of vestibular organs, nerves, or the brain [37, 38]. It has also been reported that cerebellar ischemia, chronic vertigo, acute dizziness, and balance disorders may be the first signs of severe neurological disorders related to the vestibular and ocular motor systems [39]. Previc [40] identified a few of the risk factors of AD that are also risk factors for vestibular disease: aging, cerebrovascular deficiencies, diabetes and other metabolic disorders, depression, and traumatic brain injury.

Hearing loss (ICD-code 389) is independently associated with lower scores on tests of memory and incidence of all-cause dementia or AD [21–23, 41, 42]. In a meta-analysis of prospective studies, Zheng et al. [18] found that people with hearing impairment faced a higher risk of developing AD than did those in a control group. Several possible mechanisms have been proposed. It has been suggested that hearing loss affects cortical processing by diverting cognitive resources away from cognitive processes, such as working memory toward auditory processing. It is also possible that social isolation due to hearing loss could contribute to the development of AD. There is also the strong possibility that both diseases have a common cause and that hearing loss is simply an early condition indicative of the underlying pathology [6, 43–46].

Permanent or sudden dysfunctions in the sensory system (e.g., sensorineural hearing loss) can lead to affective disorders, including depression, anxiety, and bipolar disorder [47]. The findings in the present study support this hypothesis. The long-term impairment of communication pathways can impose considerable strain on patients, undermining their quality of life, leading to social isolation, and compromising their ability to engage in pleasurable activities [48]. The risk intensity associated with affective disorders depends on the duration of sensory loss [49]. Vestibular dysfunction (a type of sensory loss) has been associated strongly with the concurrence of symptoms of affective disorders. This suggests the presence of vestibular cognitive affective syndrome [50]; however, vestibular dysfunction is related more directly to memory impairment. In the present study, mental disorders (ICD-codes 300, 307, 296, and 311) as well as hearing and vertiginous syndromes (ICD-codes 389 and 386) appeared simultaneously at least 4- to 9- years before the first diagnosis of LOAD.

Functional digestive disorders (FGIDs, ICD-code 564) exhibited a direct risk effect for LOAD development in this study. Irritable bowel syndrome (IBS) is a type of functional gastrointestinal disorder (FGID). Using the Taiwan NHIRD, Chen et al. [28] found that a new diagnosis of IBS had a positive effect on LOAD incidence and that this effect was obvious only in patients who were ≥ 50 years old. They suggested that the gut-brain axis (GBA) may play an important role in the association between IBS and AD. The GBA has recently been linked to cognitive performance, behavior, and emotions [51]. GBA is a bidirectional communication system comprising neural pathways encompassing the autonomic nervous system (ANS), enteric nervous system (ENS), immune system, and neuroendocrine system (such as acetylcholine, dopamine, 5 HT, and serotonin) [52], especially including the hypothalamic–pituitary–adrenal axis (HPA axis). The expansion of the GBA to include the functions of gut flora is referred to as the microbiome–gut–brain axis. Researchers have demonstrated that neuroinflammation can be triggered by pathogenic gut microbiota [53]. These, as well as a dysfunctional GBA, may promote cognitive impairment. It has been posited that interactions between dysfunctional GBA and the CNS can be attributed to changes in brain chemistry and neuro-endocrine systems involved in anxiety, depressive-like behaviors, stress response, and memory function [54]. In clinical practice, IBS is treated as a microbiome-GBA disorder [55]. The continual triggering of neuroinflammation by the gut has been shown to cause damage in various regions of the brain [56] and increase the risk of developing neurodegenerative disorders including AD [57].

Disorders of the urethra and urinary tract (ICD-code 599) include functional urogenital tract disorders, such as urethral hypermobility, urinary tract infection, and urethral instability. One review [58] proposed the existence of a “bladder–gut–brain axis (BGBA)” to explain the frequent co-occurrence of functional urological and gastrointestinal disorders. This interaction across organ systems could perhaps be attributed to an underlying central hypersensitivity manifesting as bodily distress. Within this schema, psychological and physical stress pathways could easily generate a range of false alarms and could arise the co-occurrence of functional disorders and mental conditions. In the present study, disorders of the urethra and urinary tract (ICD-code 599) as well as functional digestive disorders (ICD-code 564) were found to occur simultaneously at least 1 to 8 years prior to the first diagnosis of LOAD.

### Limitation

One substantial limitation of this study (as is the case with other studies that use routine data) is the limited amount of information related to other potential confounders, such as body mass index, diet pattern, blood pressure, blood sugar, smoking, family history, mood disorders, and therapy for diabetes. It is possible that disease onset and diagnosis may differ according to the economic status and residence of patients, due to the fact that these variables can affect access to neurologists. Nonetheless, the association of some conditions (e.g., anxiety, depression, disorder of the vestibular system, concussion, and hearing loss) are consistent with the findings in many previous studies. Our use of path analysis also identified a number of relevant diseases (e.g., functional digestive disorders) that have scarcely been addressed in previous studies.

## Conclusions

This study discovered that a number of conditions occurring prior to LOAD onset (e.g., mental conditions, hearing, vestibular system, and functional digestive disorder) may play an important role in the pathogenesis of LOAD. Moreover, our corrected pathways among neuropsychiatric conditions, the digestive system, and LOAD support the assertion that the gut–brain axis may play important roles in the pathogenesis of LOAD. Future studies will be needed to elucidate the mechanism underlying these associations.

## Supporting information

S1 Fig Prior four-year pathway model: Positive effects on LOAD incidence. Codes listed in the International Classification of Diseases, Ninth Revision, are displayed in the box.
(DOCX)

S2 Fig Prior four-year pathway model: Negative effects on LOAD incidence. Codes listed in the International Classification of Diseases, Ninth Revision, are displayed in the box. The green box denotes the predictors that have negative effects on LOAD.
(DOCX)

S1 Table Indices related to overall goodness-of-fit of the three models during the four years prior to the first diagnosis of LOAD. (DOCX)
