# Supplementary material for: Exploring prior diseases associated with incident late-onset Alzheimer’s disease dementia
Source: PLoS One. 2020 Jan 24;15(1):e0228172. doi: 10.1371/journal.pone.0228172 (PMC6980504; doi:10.1371/journal.pone.0228172)
Supplement: S1 Fig — Codes listed in the International Classification of Diseases, Ninth Revision, are displayed in the box. (DOCX) [file pone.0228172.s001.docx]

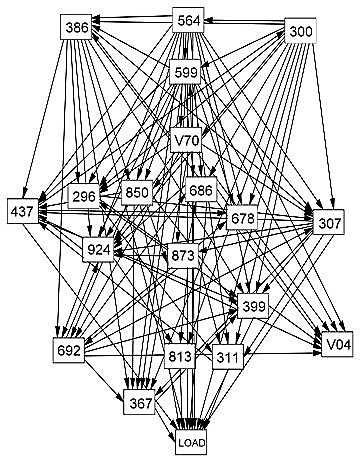


**S1 Fig. Prior four-year pathway model: Positive effects on LOAD incidence.** Codes listed in the International Classification of Diseases, Ninth Revision, are displayed in the box.
