# Supplementary material for: Exploring prior diseases associated with incident late-onset Alzheimer’s disease dementia
Source: PLoS One. 2020 Jan 24;15(1):e0228172. doi: 10.1371/journal.pone.0228172 (PMC6980504; doi:10.1371/journal.pone.0228172)
Supplement: S1 Table — (DOCX) [file pone.0228172.s003.docx]

**S1 Table. Indices related to overall goodness-of-fit of the three models during the four years prior to the first diagnosis of LOAD.**

| Indicator | Positive model | Negative model | Final model | Acceptable Values |
| --- | --- | --- | --- | --- |
| SRMR | 0.01 | 0.01 | 0.07 | <0.08 |
| RMSEA | 0.01 | 0.00 | 0.00 | <0.08 |
| CFI | 0.99 | 1.00 | 1.00 | >0.9 |
| GFI | 0.99 | 1.00 | 0.99 | >0.9 |
| NFI | 0.98 | 1.00 | 0.96 | >0.9 |

SRMR: Standardized root mean square residual; RMSEA: Root mean square error of approximation; CFI: Comparative fit Index; GFI: Goodness-of-fit statistic; NFI: Normed-fit Index
